# Variation in left ventricular cardiac magnetic resonance normal reference ranges: systematic review and meta-analysis

**DOI:** 10.1093/ehjci/jeaa089

**Published:** 2020-05-27

**Authors:** Zahra Raisi-Estabragh, Asmaa A M Kenawy, Nay Aung, Jackie Cooper, Patricia B Munroe, Nicholas C Harvey, Steffen E Petersen, Mohammed Y Khanji

**Affiliations:** 1 William Harvey Research Institute, NIHR Barts Biomedical Research Centre, Queen Mary University of London, Charterhouse Square, London EC1M 6BQ, UK; 2 Barts Heart Centre, St Bartholomew’s Hospital, Barts Health NHS Trust, West Smithfield, London EC1A 7BE, UK; 3 MRC Lifecourse Epidemiology Unit (MRCLEU), Tremona Rd, Southampton SO16 6YD, UK; 4 NIHR Southampton Biomedical Research Centre, University of Southampton and University Hospital Southampton NHS Foundation Trust, Tremona Road, Southampton, SO16 6YD, UK

**Keywords:** cardiac magnetic resonance, reference range, normal range, left ventricle

## Abstract

**Aims:**

To determine population-related and technical sources of variation in cardiac magnetic resonance (CMR) reference ranges for left ventricular (LV) quantification through a formal systematic review and meta-analysis.

**Methods and results:**

This study is registered with the International Prospective Register of Systematic Reviews (CRD42019147161). Relevant studies were identified through electronic searches and assessed by two independent reviewers based on predefined criteria. Fifteen studies comprising 2132 women and 1890 men aged 20–91 years are included in the analysis. Pooled LV reference ranges calculated using random effects meta-analysis with inverse variance weighting revealed significant differences by age, sex, and ethnicity. Men had larger LV volumes and higher LV mass than women [LV end-diastolic volume (mean difference = 6.1 mL/m^2^, *P*-value = 0.014), LV end-systolic volume (MD = 4 mL/m^2^, *P*-value = 0.033), LV mass (mean difference = 12 g/m^2^, *P*-value = 7.8 × 10^−9^)]. Younger individuals had larger LV end-diastolic volumes than older ages (20–40 years vs. ≥65 years: women MD = 14.0 mL/m^2^, men MD = 14.7 mL/m^2^). East Asians (Chinese, Korean, Singaporean-Chinese, *n *=* *514) had lower LV mass than Caucasians (women: MD = 6.4 g/m^2^, *P*-value = 0.016; men: MD = 9.8 g/m^2^, *P*-value = 6.7 × 10^−5^). Between-study heterogeneity was high for all LV parameters despite stratification by population-related factors. Sensitivity analyses identified differences in contouring methodology, magnet strength, and post-processing software as potential sources of heterogeneity.

**Conclusion:**

There is significant variation between CMR normal reference ranges due to multiple population-related and technical factors. Whilst there is need for population-stratified reference ranges, limited sample sizes and technical heterogeneity precludes derivation of meaningful unified ranges from existing reports. Wider representation of different populations and standardization of image analysis is urgently needed to establish such reference distributions.

## Graphical Abstract

**Figure:**
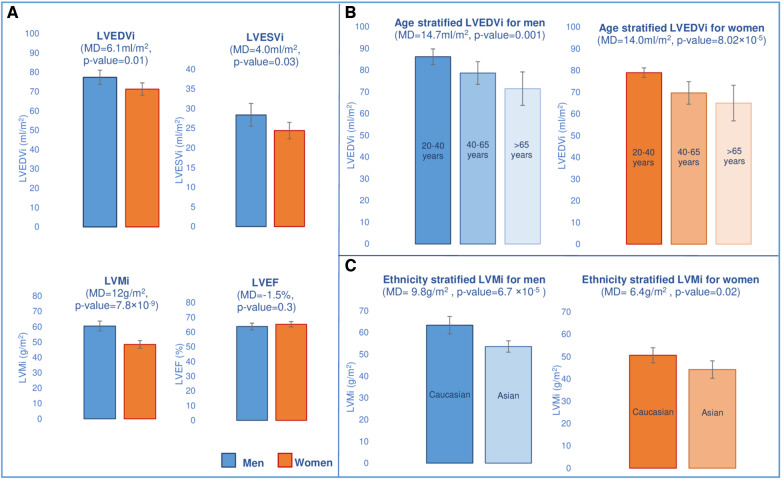

## Introduction

Accurate quantification of left ventricular (LV) structure and function is key to clinical decision making in cardiology. LV cavity volumes in end-systole (LVESV) and end-diastole (LVEDV) reflect adverse myocardial remodelling.^1^ LV mass (LVM), is an independent prognostic marker in individuals with and without cardiovascular disease.^2–4^ LV ejection fraction (LVEF) provides an estimate of LV systolic function and is the determinant of many important clinical decisions such as cardiac-resynchronization therapy, valve interventions, and management of heart failure syndromes.^5–8^

Cardiac magnetic resonance (CMR) imaging is the reference test for cardiac chamber quantification and is increasingly used to guide difficult clinical decisions. However, there is lack of consensus on normal reference ranges with variation in published reports.^9^ Whilst there are known sex, age, and ethnic differences in cardiac morphology,^10^^,^^11^ these differences have not been adequately studied with CMR and commonly quoted reference ranges are based on small cohorts that do not always represent the populations to which they are applied.

Previous attempts to pool results from different CMR reference ranges were limited by the datasets available at the time, with small sample sizes, inability to provide age and ethnicity stratification, or perform a formal meta-analysis.^12^ In the last 5 years, there has been a surge of publications reporting normal CMR reference ranges from around the world. The objective of this study is to determine population-related (sex, age, and ethnicity) and technical sources of heterogeneity through a formal systematic review and meta-analysis of published CMR reference ranges.

## Methods

This study is registered online with the International Prospective Register of Systematic Reviews (PROSPERO, https://www.crd.york.ac.uk/PROSPERO/; registration number: CRD42019147161, 27 April 2020, date last accessed). Methods are in accordance with the PRISMA statement (Transparent Reporting of Systematic Reviews and Meta-Analyses, http://prisma-statement.org/, 27 April 2020, date last accessed). The PRISMA checklist is provided in the Supplementary data online.

### Selection criteria

We selected studies that defined a normal reference range in healthy adults (>18 years-old) with sample sizes of ≥50, reported in the English language. We required confirmation of healthy status of participants. We accepted studies with 1.5-T or 3-T scanners from all vendors. We restricted to studies using steady state free precession (SSFP) sequences, as this reflects current clinical standards for volume quantification. We required LV quantification to be made using short-axis cine images using a predefined standard operating procedure for image acquisition and analysis. Studies selected for quantitative analysis were required to report sex-stratified LVM, LVEDV, LVESV, and LVEF in a manner where mean and standard deviation values in indexed formats [indexed to body surface area (BSA), denoted by *i*] could be extracted.

### Search strategy

Z.R.E. and A.A.M.K. independently searched Ovid Medline (1946–April 2019) and Embase electronic databases. Relevant subject headings were used to conduct the search using MeSH terms (Medical Subject Headings) for Medline and the equivalent, Emtree, for Embase. Subject headings and their ‘trees’ were examined, and relevant subheadings were selected, related terms were included using the explode command (Supplementary data online, *Table S1*). Search terms were combined using Boolean operators. Selected terms were included in the search as keywords. We performed separate keyword search of titles and abstracts to ensure capture of newer publications not yet incorporated into MeSH/Emtree classifications. The final output was limited to studies in adults (>18 years-old) and in the English language.

### Study selection

Study selection was through a process of title screening, abstract review, and full-text review carried out independently by A.A.M.K. and Z.R.E. At each iteration, results were merged and duplicates removed. Further studies were identified through reference and author searching. Decision for study eligibility was based on predefined selection criteria. In case of disagreement, decisions were taken through discussion after review of full text and mediation by M.Y.K.

### Quality assessment

As this review was not based on intervention-outcome studies, existing quality assessment tools were not entirely applicable. We therefore designed a quality assessment protocol tailored to our purpose based on revised elements from the ROBINS-I (The Risk Of Bias In Non-randomized Studies-of Interventions) and QUADAS-2 (Quality Assessment of Diagnostic Accuracy Studies) assessment tools.^13^^,^^14^

### Data extraction

Mean, standard deviation, and sample size for sex-stratified LVM indexed (LVMi), LVESV indexed (LVESVi), LVEDV indexed (LVEDVi), and LVEF were extracted from individual studies. Data extraction was carried out independently by Z.R.E. and A.A.M.K. and cross-checked by Z.R.E.

### Statistical analysis

Statistical analysis was with the ‘meta: General package for meta-analysis’ package on the R studio platform [R Core Team (2018). R: A language and environment for statistical computing. R Foundation for Statistical Computing, Vienna, Austria. URL https://www.R-project.org/, 27 April 2020, date last accessed].^15^ We calculated pooled age, sex, and ethnicity-specific ranges for LV parameters indexed to BSA. We used random effects meta-analysis of single means with inverse variance weighting to calculate pooled values. Between-study heterogeneity was assessed with τ,^2^*I*^2^, Q statistic, and the related *P*-value. For subgroup analysis, mean difference (MD), Q statistic, and *P*-values are presented. We performed sensitivity analysis with the following variables: scanner vendor, magnet strength, post-processing analysis software, papillary muscles contouring (inclusion/exclusion in LVM). To assess the impact of the larger studies in the meta-analysis on the overall results, we display results for both fixed and random effects models in the figures. A large study with extreme results would lose influence under the random effects model. Our analyses demonstrate similar estimates from fixed and random effects models; therefore we conclude that variations in study sample size are not having a disproportionate impact on the results. For further illustration, we performed sensitivity analysis with exclusion of the two largest studies from the pooled estimates, which also did not significantly alter the pooled estimates.

## Results

### Systematic review

**Figure 1:**
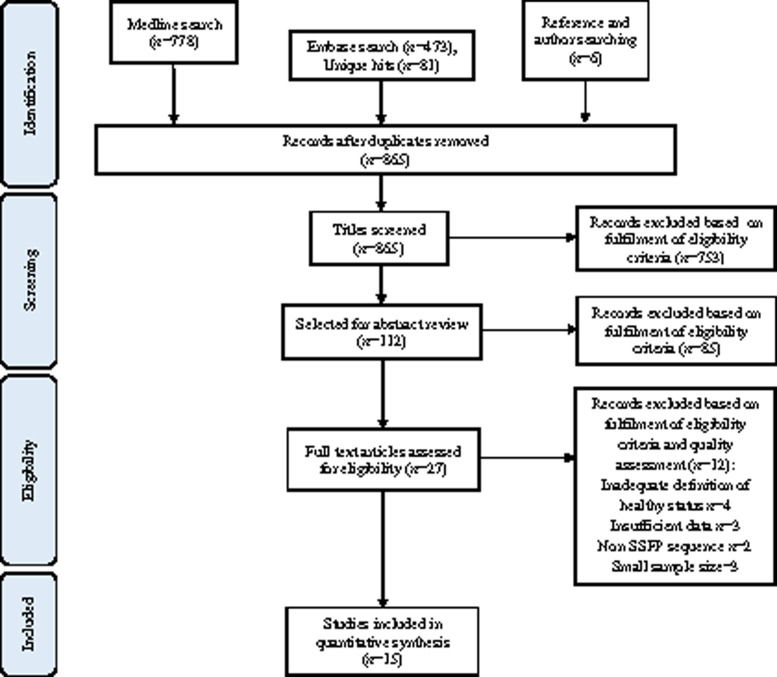
Flow diagram summarizing flow of information through different phases of the systematic review.

Our approach is summarized in the PRISMA flow diagram (*Figure 1*). Combined Ovid Medline and Embase searches yielded 859 unique hits; 6 additional citations were obtained from cross-referencing and author searches. After title screening, 112 citations were deemed potentially relevant and selected for abstract review. From these, 27 papers were selected for full-text review based on fulfilment of the inclusion criteria. A further 12 studies were excluded after examination of the full text based on quality assessment and consideration of inclusion criteria. Fifteen studies were selected for inclusion in the meta-analysis. Of these, two did not report LVESV, therefore, 13 studies are included in analysis for this parameter.

### Quality assessment

Pertinent quality indicators were systematically assessed for studies selected for full-text review. There were differences in the definition of ‘healthy status’ with variable use of clinical assessment, blood tests, and non-invasive tests (echocardiography and electrocardiogram) to exclude disease. There were also variations in the number of readers and reports of inter-/intra-observer variability. Overall, the studies included in the meta-analysis are of high quality with clearly defined study objectives and imaging protocols (Supplementary data online, *Table S2*).

### Summary of selected studies

Overall 2132 women and 1890 men from 15 studies published between 2003 and 2018 are included in the analysis (*Table 1*). The age range is between 20 and 91 years. There are five studies from non-Caucasian cohorts: two from Chinese populations,^17^^,^^21^ and one study each from Singaporean-Chinese,^20^ Korean,^25^ and Brazilian^24^ cohorts. There are 10 studies from Caucasian populations.^16^^,^^18^^,^^19^^,^^22^^,^^23^^,^^26–30^ Both the Chinese studies and the study from Singapore use a 3-T scanner, all others use 1.5-T scanners. Scanners included several Siemens and Philips models; one of the earlier studies used a General Electric (GE) scanner. Various versions of a wide range of post-processing software packages were used for endocardial contouring. Contouring technique was either manual or semi-automated with manual edits. Eleven studies included papillary muscles in the LVM, the remainder as part of the blood pool.

**Table 1:** Summary of studies selected for inclusion in the meta-analysis

Author, year of publication	Single/ multicentre	Sample size, male:female	Age^a^	Ethnicity	Scanner vendor	Field strength (T)	Analysis software	Contouring technique	Papillary muscle included/excluded from LVM
Bulow *et al*.,^16^ 2018	Single	*n* = 617	Men (43)	Caucasian	Siemens Magnetom	1.5	QMass MR, Medis	Manual	Included
		291:326	Women (45)						
Lei *et al*.,^17^ 2017	Single	*n* = 120	20–83	Chinese	Siemens Magnetom	3	QMass MR, Medis	Manual	Excluded
		60:60							
Petersen *et al*.,^18^ 2017	Single	*n* = 800	45–74	Caucasian	Siemens Magnetom	1.5	CMR42, Circle Cardiovascular Imaging	Manual	Excluded
		368:432							
Aquaro *et al*.,^19^ 2017	Multi	*n* = 255	15–80	Caucasian	Multi-vendor	1.5	Multiple	Manual	Included
		140:115							
Le *et al*.,^20^ 2016	Single	*n* = 180	20–69	Singaporean-Chinese	Philips Ingenia	3	CMR42, Circle Cardiovascular Imaging	Not stated	Included
		91:89							
Li *et al*.,^21^ 2016	Single	*n* = 90	40–65	Chinese	Philips Achieva	3	Philips Medical Systems, Philips	Manual	Included
		45:45							
Le Ven *et al*.,^22^ 2016	Single	*n* = 434	18–35	Caucasian	Philips Achieva	1.5	CMR42, Circle Cardiovascular Imaging	Semi-automated	Included
		196:238							
Yeon *et al*.,^23^ 2015	Single	*n* = 852	Men (61)	Caucasian	Philips Gyroscan	1.5	EasyVision 5.1, Philips	Manual	Excluded
		340:512	Women (62)						
Macedo *et al*.,^24^ 2013	Multi	*n* = 107	20–80	Brazilian	Philips Achieva	1.5	Multiple	Semi-automated	Included
		54:53							
Chang *et al*.,^25^ 2012	Single	*n* = 124	20-70	Korean	Siemens Magnetom	1.5	Argus, Siemens	Manual	Excluded
		64:60							
Teo *et al*.,^26^ 2008	Single	*n* = 60	51	Non-aboriginal Australian (Caucasian)	Siemens Sonata	1.5	Argus, Siemens	Manual	Included
		41:19							
Maceira *et al*.,^27^ 2006	Single	*n* = 120	20–80	Caucasian	Siemens Sonata	1.5	CMRtools, Cardiovascular Imaging Solutions	Semi-automated	Included
		60:60							
Nikitin *et al*.,^28^ 2006	Single	*n* = 95	22–91	Caucasian	General Electric SignaCV/i	1.5	MRI-MASS, Medis	Semi-automated	Included
		47:48							
Hudsmith *et al*.,^29^ 2005	Single	*n* = 108	21–68	Caucasian	Siemens Sonata	1.5	Argus, Siemens	Manual	Included
		63:45							
Alfakih *et al*.,^30^ 2003	Single	*n* = 60	20–65	Caucasian	Philips Intera	1.5	MRI-MASS, Medis	Manual	Included
		30:30							

LVM, left ventricular mass; T, Tesla; *n* denotes total sample size available for analysis.

a Age: range, or mean (years).

### Meta-analysis

#### Stratification by sex

**Figure 2:**
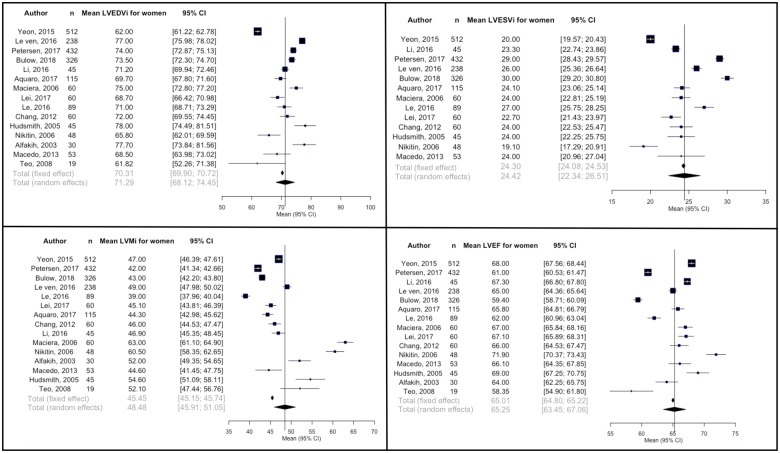
Forest plots of left ventricular parameters indexed to body surface area for women.^a^ Both fixed effect and random effects estimates are presented. The vertical reference line corresponds to random effects pooled mean estimate. CI, confidence interval; LVEDVi, left ventricular end-diastolic volume indexed to body surface area (mL/m^2^); LVEF, left ventricular ejection fraction (%); LVESVi, left ventricular end-systolic volume indexed to body surface area (mL/m^2^); LVMi, left ventricular mass indexed to body surface area (g/m^2^).

**Figure 3:**
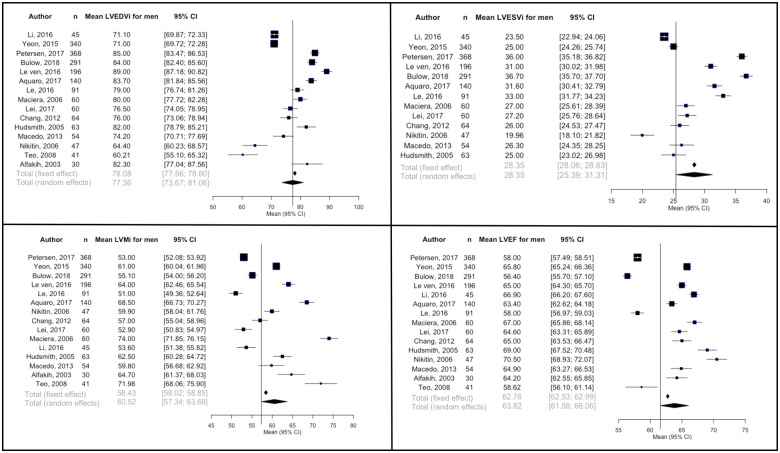
Forest plots of left ventricular parameters indexed to body surface area for men.^a^ Both fixed effect and random effects estimates are presented. The vertical reference line corresponds to random effects pooled mean estimate. CI, confidence interval; LVEDVi, left ventricular end-diastolic volume indexed to body surface area (mL/m^2^); LVEF: left ventricular ejection fraction (%); LVESVi, left ventricular end-systolic volume indexed to body surface area (mL/m^2^); LVMi, left ventricular mass indexed to body surface area (g/m^2^).

Results for sex-stratified analyses are summarized in *Table 2*, *Figures 2* and *3, and Panel A* of the graphical abstract. Compared to women, men had significantly larger LVEDVi (MD = 6.1 mL/m^2^, *P*-value = 0.014), LVESVi (MD = 4.0 mL/m^2^, *P*-value = 0.033), and LVMi (MD = 12.0 g/m^2^, *P*-value = 7.8 × 10^−9^). LVEF was not significantly different between men and women (MD = −1.5%, *P*-value 0.33). In both men and women, there was significant between-study heterogeneity for all LV parameters (*I*^2^ > 97% for all).

**Table 2:** Pooled mean left ventricular parameters with sex stratification and expression of between-study and subgroup heterogeneity

			Between study heterogeneity				Subgroup differences (men vs. women)		
		Mean (95% CI)	Q statistic	τ ^2^	*I* ^2^	*P*-value	Mean difference	*Q* statistic	*P*-value
LVEDVi (mL/m^2^)	Men	77.4 (73.7–81.1)	655.4	51.1	97.9%	8.5 × 10^−131^	6.1	6.0	0.01
	Women	71.3 (68.1–74.5)	736.4	36.6	98.1%	4.3 × 10^−148^			
LVESVi (mL/m^2^)	Men	28.4 (25.4–31.3)	1196.4	29.1	99.0%	1.0 × 10^−248^	4.0	4.5	0.03
	Women	24.4 (22.3–26.5)	933.7	14.3	98.7%	3.3 × 10^−192^			
LVMi (g/m^2^)	Men	60.5 (57.3–63.7)	773.9	18.2	98.2%	4.3 × 10^−156^	12	33.3	7.8 ×10^−9^
	Women	48.5 (45.9–51.0)	942.4	24.7	98.5%	3.5 × 10^−192^			
LVEF (%)	Men	63.8 (61.6–66.1)	1272.2	19.2	98.9%	5.1 × 10^−263^	−1.5	1.0	0.3
	Women	65.3 (63.5–67.1)	960.2	12.1	98.5%	5.5 × 10^−196^			

Significance level is set at *P*-value <0.05. Random effects model is used for assessment of subgroups and between-study heterogeneity.

CI, confidence interval; LVEDVi, left ventricular end-diastolic volume indexed to body surface area; LVEF, left ventricular ejection fraction; LVESVi, left ventricular end-systolic volume indexed to body surface area; LVMi, left ventricular mass indexed to body surface area.

#### Stratification by age and sex

Three age categories were created to represent young (20–40 years), middle-aged (40–65 years), and older (≥65 years) adults. These age cut-offs allowed inclusion of the largest possible pooled sample from all studies. Age- and sex-stratified results are presented in Supplementary data online, *Table S3*. Both men and women had significantly larger LVEDVi in younger age (Supplementary data online, *Figure S1*) with similar magnitude of difference (20–40 years vs. ≥65 years: women MD = 14.0 mL/m^2^, men MD = 14.7 mL/m^2^). A trend for larger LVESVi in younger age is observed for both men and women but is not statistically significant in either. There were non-significant trends towards greater LVMi in younger and higher LVEF in older individuals. The data available did not permit analysis with age as a continuous measure or with more granular age bands. Between-study heterogeneity remained high after sex and age stratification.

#### Stratification by sex and ethnicity

Pooled values were calculated for two ethnicity categories: East Asian (Chinese, Singaporean-Chinese, Korean) and Caucasian (including non-Aboriginal Australian). East Asian men and women had significantly lower LVMi compared to Caucasians (women: MD = 6.4 g/m^2^, *P*-value = 0.016; men: MD = 9.8 g/m^2^, *P*-value = 6.7 × 10^−5^), this difference was more consistent and of greater magnitude in men (Supplementary data online, *Figure S2*). Further comparison was made between pooled values for Caucasians, East Asians, and the one Brazilian cohort. Again, significant subgroup differences were observed in LVMi for both men and women. Brazilian men and women had greater LVMi than East Asians, but lower values than Caucasians. There were no significant ethnic differences in any of the other LV parameters (Supplementary data online, *Table S4*). We present pooled sex-stratified results for Caucasians and East Asians with addition of age stratification for Caucasians (*Figures 5 and**6*) We are unable to provide pooled age- and sex-stratified results for East Asians due to variation in age bands and reporting of stratified results in the original studies. There was high statistical heterogeneity between studies despite these population stratifications.

**Figure 4:**
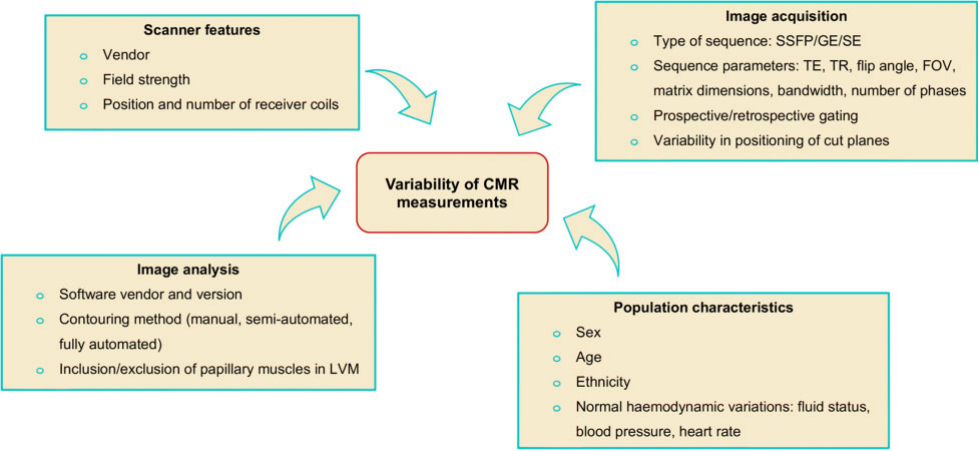
Potential sources of variability in cardiac magnetic resonance measurements. CMR, cardiac magnetic resonance; FOV, field of view; GE, gradient echo; LVM, left ventricular mass; SE, spin echo; SSFP, steady state free precession; TE, echo time; TR, repetition time.

**Figure 5:**
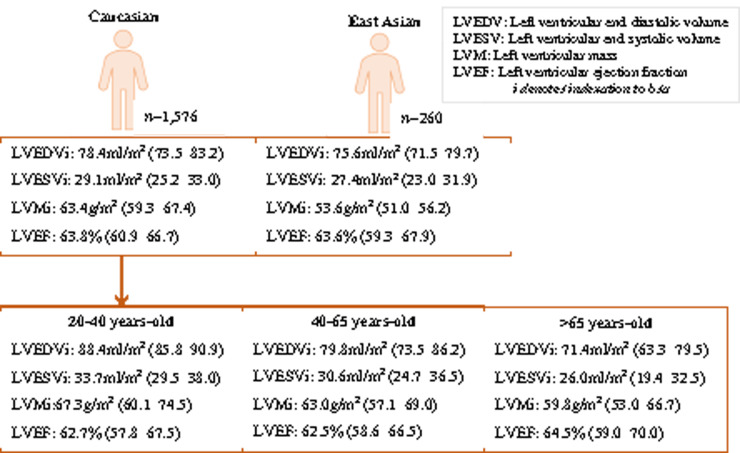
Pooled mean (95% CI) left ventricular parameters for men, stratified by age and ethnicity. Results are pooled random effects means with corresponding 95% CIs. LVEDVi, left ventricular end-diastolic volume indexed to body surface area (mL/m^2^); LVEF, left ventricular ejection fraction (%); LVESVi, left ventricular end-systolic volume indexed to body surface area (mL/m^2^); LVMi, left ventricular mass indexed to body surface area (g/m^2^).

**Figure 6:**
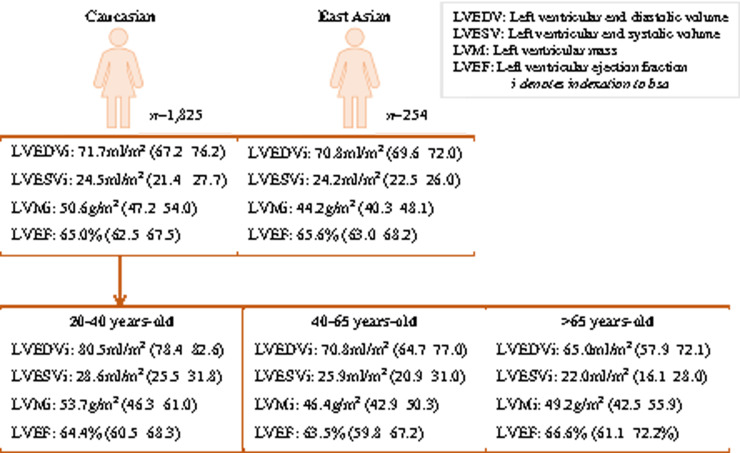
Pooled mean (95% CI) left ventricular parameters for women, stratified by age and ethnicity. Results are pooled random effects means with corresponding 95% CIs. LVEDVi, left ventricular end-diastolic volume indexed to body surface area (mL/m^2^); LVEF, left ventricular ejection fraction (%); LVESVi, left ventricular end-systolic volume indexed to body surface area (mL/m^2^); LVMi, left ventricular mass indexed to body surface area (g/m^2^).

#### Sensitivity analyses

To explore potential technical sources of between-study heterogeneity, sensitivity analyses were performed with the following variables: scanner vendor, field strength, post-processing software, and papillary muscle contouring (included vs. excluded from LVM) (Supplementary data online, *Table S5*). Studies including papillary muscles as part of the LVM reported significantly higher LVM for both men (MD = 7.1 g/m^2^, *P*-value = 0.017) and women (MD = 6.0 g/m^2^, *P*-value = 0.029). Despite stratification for sex and contouring methodology, heterogeneity between studies remained high, with greater heterogeneity for studies contouring papillary muscles as part of LVM (Supplementary data online, *Figure S3*). The post-processing software used for contouring also impacted results with Argus software from Siemens Medical yielding significantly smaller LVESVi and higher LVMi in comparison to other post-processing tools. We also note a significant relationship between lower LVMi and 3-T field strength scanners. Limited samples and significant methodological heterogeneity at all levels meant that pooling of results with stratification for multiple technical and population-related factors was not possible.

## Discussion

### Summary of findings

We present the first formal systematic review and meta-analysis of CMR normal reference ranges incorporating results from 1890 men and 2132 women from 15 studies. Pooled results demonstrate significant differences in LV parameters by sex, age, and ethnicity. Compared to women, men had larger cavity volumes and greater LVMi. Younger individuals had larger LV volumes, higher LVMi and lower LVEF in comparison to older ages. Individuals with East Asian ancestry had lower LVMi in comparison to Caucasians. Between-study heterogeneity was high for all parameters despite stratification for population-related factors. Sensitivity analyses identified differences in contouring methodology, post-processing software, and magnet field strength as potential significant contributors to the observed between-study heterogeneity. Limited sample sizes from existing results and methodological variation at all levels precludes recommendation of robust unified reference ranges from this analysis.

### Comparison with previous literature

The observed sex, age, and ethnic differences in LV measures are consistent with previous reports using cardiac computed tomography, echocardiography, and gradient echo CMR.^31–35^ Echocardiography studies report important differences in cardiac morphology of healthy individuals of South Asian and Afro-Caribbean ethnicity in comparison to Caucasians.^32^^,^^36^^,^^37^ Furthermore, there are reports of differential significance of alterations in LV parameters in different ethnic populations. For instance, Akintoye *et al*. report greater prognostic utility of LVMi for predicting cardiovascular events for Chinese and Hispanic populations in comparison to non-Hispanic Whites.^38^ Similarly, there are reports of significant ethnic differences in ventricular remodelling in response to important cardiovascular risk factors such as hypertension.^36^ As ethnic variation exist for LV parameters, it is possible that there are also ethnic differences in the morphology of other cardiac chambers (right ventricle, atria). Whilst in recent years, there have been reports of CMR references ranges from several non-Caucasian cohorts, data from a wide range of ethnicities remains absent, as such, our understanding of ethnic differences in CMR derived measures of cardiac morphology remains incomplete.

In addition to the expected variations by population-related factors, we identified important technical sources of heterogeneity. We identified magnet strength (3 T vs. 1.5) as a significant source of variation, in particular lower LVMi reported by the studies using 3 T scanners. Certainly, it is conceivable that higher spatial resolution produced by expert programming of pulse sequences with 3 T scanners provides superior endocardial border definition and thus more accurate contouring of the LV endocardium with exclusion of an intracavity trabecular layer that may be included within LVM at lower spatial resolutions. However, there are other factors that need consideration. For instance, the 3 T studies are all more recent publications (2016 onwards), image analysis for these studies has been conducted using modern post-processing software perhaps allowing for more accurate endocardial border contouring in comparison to older studies. There are also important population factors—all the studies with 3 T scanners are from East Asian cohorts, whereas all studies on Caucasians are with 1.5 T scanners. With the presence of multiple overlapping variables, it is impossible to isolate definitively the effect of 3 T vs. 1.5 T in this study. Previous studies dedicated to comparison of LV measures at 3 T vs. 1.5 T have not shown significant differences between the two.^39^ On balance, our judgement is that the observed differences are more likely related to ethnic differences with perhaps a smaller contribution from the various technical sources of variation.

Consistent with previous reports, we identified differences in endocardial contouring as a significant source of variation.^40^^,^^41^ There was greater heterogeneity between studies that included papillary muscles within LVM compared to those that did not, perhaps reflecting difficulties in reproducibly tracing the irregular geometry of papillary muscles. Previous studies report similar variations with the potential for clinically important differences in the assessment of relevant pathologies such as hypertrophic cardiomyopathy and Fabry’s disease.^42^^,^^43^ Whilst other sources of technical variation do exist and perhaps have a cumulative effect, it does seem that contouring technique is the most important. Interestingly, a small study of variation of CMR derived LV measures from the use of different software packages demonstrated no significant variation from the software programmes with the application of a standardized contouring protocol and a single scanner vendor.^44^ This observation suggests that the variability in LV quantification measures may be eliminated, or certainly reduced, by development of uniform contouring practices.

Our analysis suggests that the high between-study heterogeneity is a result of cumulative effects from multiple population-related and technical sources of variation. We were unable to significantly reduce between-study heterogeneity through stratification by one or two factors and the sample size does not permit meaningful sub-analysis by greater number of variables.

### Relevance for clinical practice

Our results show that for both men and women, healthy young adults have on average 21% larger LVEDVi compared to healthy older adults (age < 40 years vs. age > 65 years: women MD = 14.0 mL/m^2^, max difference = 24.3 mL/m^2^; men MD = 14.7 mL/m^2^, max difference = 26.0 mL/m^2^). Whilst specific recommendations for age-correction cannot be made, reporting cardiologists should consider this level of variation when applying reference ranges to individuals outside represented age groups. Similar considerations should be made regarding ethnicity. Our findings show lower LVMi in East Asians compared to Caucasians with mean percentage difference of 18% and 15% in men and women, respectively (women: MD = 6.4 g/m^2^, max difference = 13.7 g/m^2^; men: MD = 9.8 g/m^2^, max difference = 16.4 g/m^2^). These differences can be clinically important. For example, consider an East Asian man with LVMi of 63 g/m^2^—whilst this is average for a Caucasian population, it is well above the upper limit of normal for Asian cohorts (56.2 g/m^2^). Where possible, ethnicity-specific reference ranges should be used. Differences produced by technical factors, in particular, contouring methodology should also be considered. For instance, our findings suggest approximately 13% greater LVMi for both men and women when contouring includes papillary muscles within LVM.

Whilst CMR remains the references standard for LV quantification, the results must be interpreted with consideration of age, sex, and ethnic differences. In addition, there are multiple technical sources of variation that may result in clinically important differences in reported values. Considering the high statistical heterogeneity between studies and the importance of technical sources of variation, we would recommend use of reference ranges that most resembles one’s own clinical practice in terms of image acquisition, analysis, and population. In cases of variation in practice from the reference range of choice, it is possible to making approximate corrections using the calculations provided here.

### Directions for future work

This work highlights the need for richer reference datasets with attention to incorporation of data from different ethnic groups and wider spectrum of ages. The lack of published data from ethnicities with known important differences in cardiac morphology is a significant limitation of existing literature. We should aim for development of reference ranges that are fully stratified by age, sex, and ethnicity. It is also important that we reduce the level of heterogeneity introduced by technical factors, with development of a unified approach to contouring methodology being a key step. However, it is difficult to make consensus recommendations at present, as it is not clear from existing literature, which contouring method best predicts clinical outcomes and/or discriminates disease. Therefore, prior to embarking on development of standardized approaches, research is needed into the prognostic and diagnostic value of different contouring methodologies. Finally, consideration of variability in cardiac morphometrics beyond traditional CMR indices is important for better understanding of differential disease patterns and risk profiles in different populations and would allow for deeper phenotyping of individuals and their disease susceptibilities.

### Limitations

Our search strategy was thorough for published reports of CMR normal reference ranges; however, we did not seek results from unpublished cohorts. Whilst this may have resulted in a larger sample size, quality control of data that has not been through a formal peer-review process is challenging and inclusion of such data may have compromised the quality of the study. There are important gaps in the literature with paucity of data for individuals in the youngest and oldest age categories and limited representation of non-Caucasian ethnicities. Our analysis reflects these gaps in published data. Whilst age, sex, and ethnicity explain part of the between-study heterogeneity, there are technical sources of variation that cannot be fully explored within the scope of this study (*Figure 4*).

## Conclusions

There is significant heterogeneity in published CMR LV reference ranges. Age, sex, and ethnicity represent significant sources of variation and we should endeavour to develop reference ranges stratified to these parameters. Different endocardial contouring methodology, scanner magnet strength, and post-processing software all contribute to the observed variability. Due to multiple sources of heterogeneity, it is not possible to produce reliable normal ranges across a wide age range, by sex or ethnicity from existing reports. Wider representation of different populations and standardization of image analysis is urgently needed to establish such reference distributions, and thus ensure global comparability of CMR measures.

**Data availability statement:** This review uses summary data from cited publications, which are retrievable by referring to the original manuscripts.

## Supplementary data

Supplementary data are available at *European Heart Journal - Cardiovascular Imaging* online.

## Funding

This work was supported by a British Heart Foundation Clinical Research Training Fellowship (FS/17/81/33318 to Z.R.E.) and was supported by a Wellcome Trust Research Training Fellowship (203553/Z/16/Z to N.A.). S.E.P. acknowledges support from the National Institute for Health Research (NIHR) Cardiovascular Biomedical Research Centre at Barts NHS Trust and has received funding from the European Union’s Horizon 2020 research and innovation programme (825903). S.E.P. acts as a paid consultant to Circle Cardiovascular Imaging Inc., Calgary, Canada and Servier. N.C.H. has received consultancy, lecture fees, and honoraria from Alliance for Better Bone Health, Amgen, MSD, Eli Lilly, Servier, Shire, Radius Health, UCB, Consilient Healthcare, Kyowa Kirin, and Internis Pharma.

**Conflict of interest:** none declared.

## Supplementary Material

jeaa089_Supplementary_Data
