# Supplementary material for: Variation in left ventricular cardiac magnetic resonance normal reference ranges: systematic review and meta-analysis
Source: Eur Heart J Cardiovasc Imaging. 2020 May 27;22(5):494–504. doi: 10.1093/ehjci/jeaa089 (PMC8081427; doi:10.1093/ehjci/jeaa089)
Supplement: jeaa089_Supplementary_Data [file jeaa089_supplementary_data.zip › Supp_Fig_1_ehj.docx]

**Supplementary Figure 1. Forest plots of age and sex stratified left ventricular end diastolic volume indexed to body surface area**


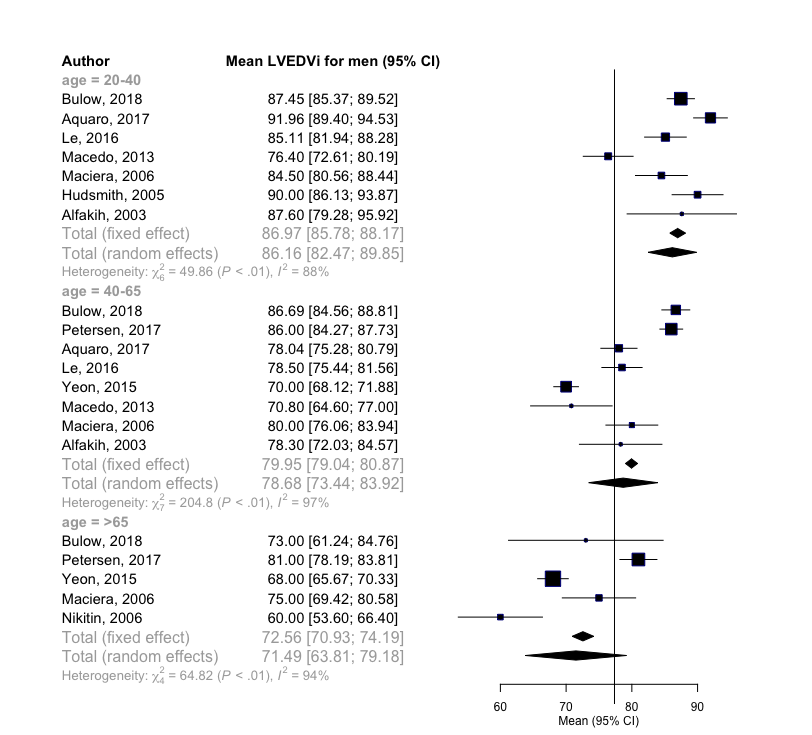

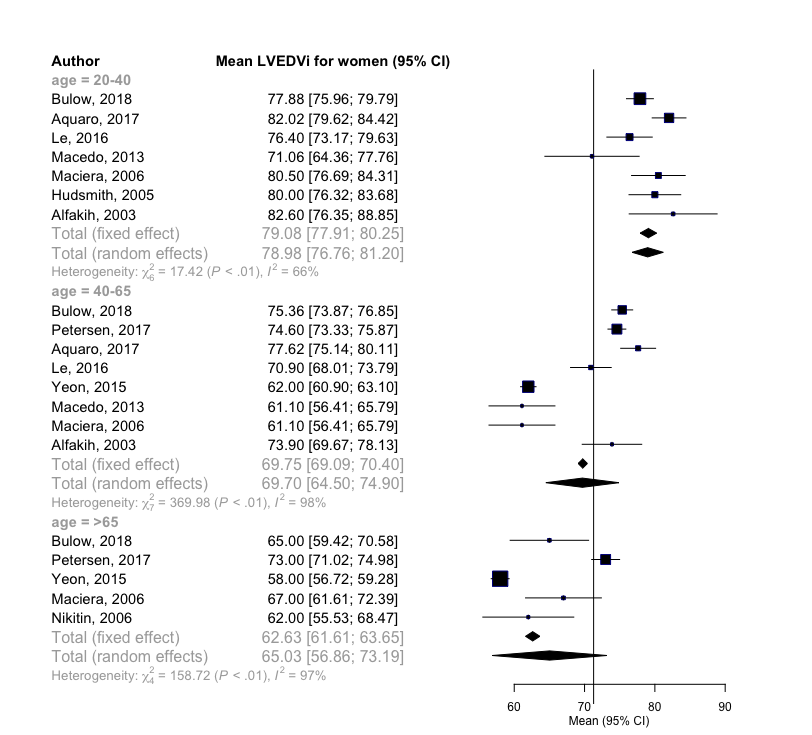


CI: confidence interval; LVEDVi: left ventricular end diastolic volume indexed to body surface area (ml/m^2^); The vertical reference line corresponds to random effects pooled mean estimate for men and women without age stratification. Vertical reference line corresponds to the pooled random effects mean for men and women without other stratification
