# Supplementary material for: Variation in left ventricular cardiac magnetic resonance normal reference ranges: systematic review and meta-analysis
Source: Eur Heart J Cardiovasc Imaging. 2020 May 27;22(5):494–504. doi: 10.1093/ehjci/jeaa089 (PMC8081427; doi:10.1093/ehjci/jeaa089)
Supplement: jeaa089_Supplementary_Data [file jeaa089_supplementary_data.zip › Supp_Fig_3_ehj.docx]

**Supplementary Figure 3. Forest plots of left ventricular mass indexed to body surface area stratified by sex and papillary muscle inclusion/exclusion in left ventricular mass**


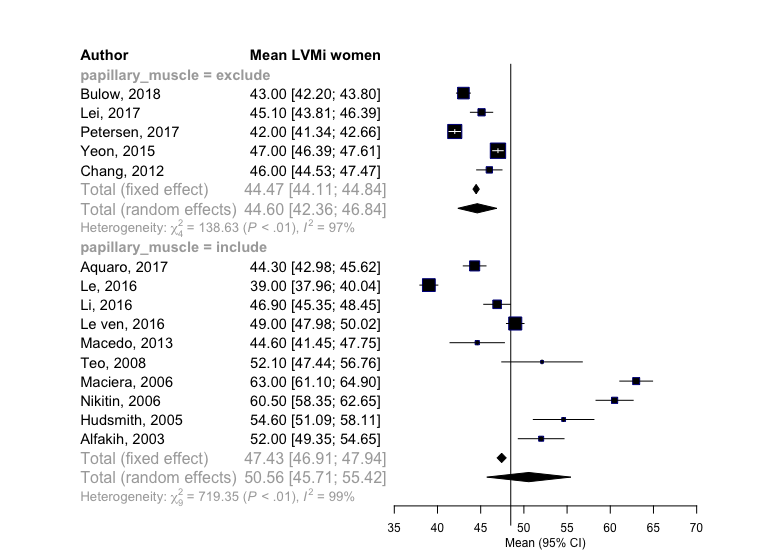

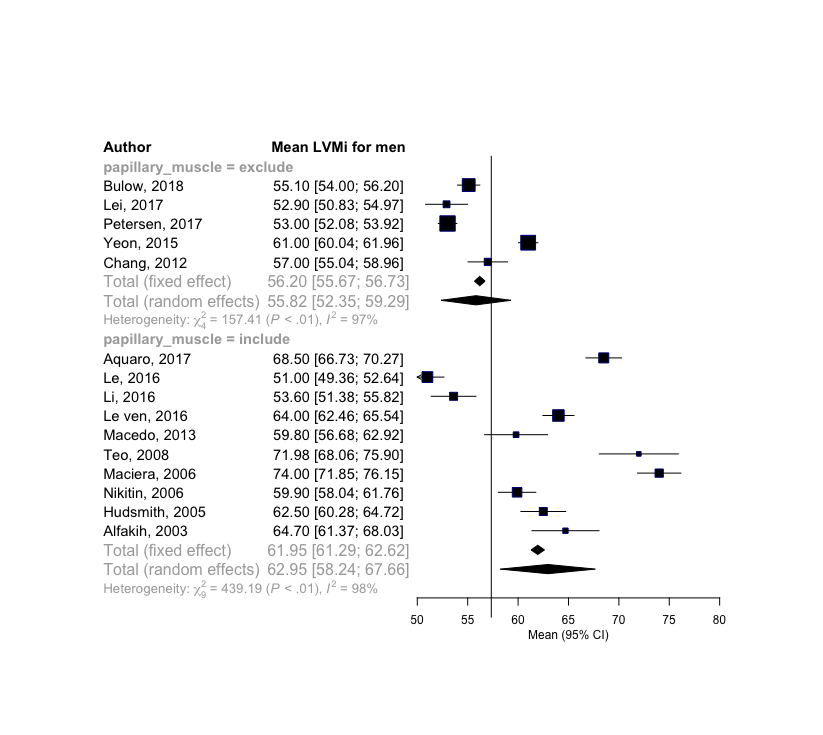


CI: confidence interval; LVMi: left ventricular end diastolic mass indexed to body surface area (g/m^2^); The vertical reference line corresponds to random effects pooled mean estimate for men and women without other stratification. Papillary_muscle = include indicates inclusion of papillary muscle in LVMi, papillary_muscle=exclude indicates exclusion of papillary muscles from LVMi.
