# Supplementary material for: Variation in left ventricular cardiac magnetic resonance normal reference ranges: systematic review and meta-analysis
Source: Eur Heart J Cardiovasc Imaging. 2020 May 27;22(5):494–504. doi: 10.1093/ehjci/jeaa089 (PMC8081427; doi:10.1093/ehjci/jeaa089)
Supplement: jeaa089_Supplementary_Data [file jeaa089_supplementary_data.zip › Supp_table1_ehj.docx]

**Supplementary Table 1. Medline search strategy**

|  | Search terms | Results (*n*) |
| --- | --- | --- |
| 1 | exp heart ventricles/ or exp myocardium/ | 245485 |
| 2 | exp cardiac volume/ or exp ventricular function, left/ or exp ventricular function, right/ | 44207 |
| 3 | 1 or 2 | 276474 |
| 4 | exp Magnetic Resonance Imaging/mt, st, sn [Methods, Standards, Statistics & Numerical Data] | 122759 |
| 5 | exp Reference Values/ | 156703 |
| 6 | normal.mp. | 1448341 |
| 7 | healthy.mp. or exp Healthy Volunteers/ | 671578 |
| 8 | 5 or 6 or 7 | 2094950 |
| 9 | 3 and 4 and 8 | 1159 |
| 10 | limit 9 to (English language and humans and "all adult (19 plus years)") | 778 |
