# Supplementary material for: Variation in left ventricular cardiac magnetic resonance normal reference ranges: systematic review and meta-analysis
Source: Eur Heart J Cardiovasc Imaging. 2020 May 27;22(5):494–504. doi: 10.1093/ehjci/jeaa089 (PMC8081427; doi:10.1093/ehjci/jeaa089)
Supplement: jeaa089_Supplementary_Data [file jeaa089_supplementary_data.zip › Supp_table2_ehj.docx]

**Supplementary Table 2. Quality assessment of studies included in the meta-analysis**

|  |  |  |  |  | **Definition of healthy status (Exclusion criteria)** | **Scanning and reporting** | | | | ***Score** |
| --- | --- | --- | --- | --- | --- | --- | --- | --- | --- | --- |
| **Author, year** | ***n*** | **Clearly defined aim** | **Data source** | **Recruitment method** |  | **Defined scanning protocol** | **Defined analysis protocol** | **Number of readers** | **Report observer variability?** |  |
| Bulow et al., 2018 | 634 | Yes | Subset of population-based study (SHIP)* | Two stage stratified cluster sampling and random cluster sampling (SHIP), subset undergoing contrast enhanced CMR included | ﻿**Cardiac disease:** MI, HF, stroke, PVD, previous cardiac surgery  **CVD risk factors:** Hypertension, diabetes  **Non-cardiac disease:** Chronic lung disease  **Medication:** cardiovascular/pulmonary medication  **Clinical assessment:** None  **Blood tests**: None  **Other investigations:** None | Yes | Yes | 2 | Inter-observer variability only | 9 |
| Lei et al., 2017 | 120 | Yes | Not stated | Prospectively recruited volunteers without known CVD | **Cardiac disease:** Any CVD  **CVD risk factors:** Hypertension  **Non-cardiac disease:** None  **Medication:** None  **Clinical assessment:** BP >140/90mmHg  **Blood tests:** Abnormal full blood count, liver/renal function  **Other investigations:** Abnormal ECG/echo | Yes | Yes | Not stated | ﻿Yes | 8 |
| Petersen et al., 2017 | 800 | Yes | Subset of population-based study (UKB) | UKB: Postal invite to all UK residents aged 20-69 years old (UKB).  This study: first 5,065 UKB participants to undergo CMR | **Cardiac disease:** Any CVD  **CVD risk factors:** Hypertension, diabetes, hyperlipidaemia, current/ex- smoker  **Non-cardiac disease:** Respiratory haematological, renal, or rheumatological disease, malignancy  **Medication:** Antihypertensives, lipid-lowering drugs, diabetic medications.  **Clinical assessment:** Chest pain or dyspnoea, BMI ≥30 kg/m^2^  **Blood tests:** None  **Other investigations:** None | Yes | Yes | 8 | Yes | 9 |
| Aquaro et al., 2017 | 255 | Yes | Not stated | Not stated | ﻿**Cardiac disease:** Any CVD  **CVD risk factors:** Hypertension, diabetes, hyperlipidaemia, smoking, drug use  **Non-cardiac disease:** Any non-cardiac illness that may affect cardiac function  **Medication:** Antihypertensives, lipid-lowering drugs  **Clinical assessment:** Abnormal physical examination, BP >149/90mmHg, family history of genetic disease, BMI >30 kg/m^2^  **Blood tests:** None  **Other investigations:** Abnormal echo/ECG | Yes | Yes | Not stated | Yes | 8 |
| Le et al., 2016 | 180 | Yes | Local community (general population) | Prospective recruitment through advertisement in local media | **Cardiac disease:** Any CVD or cerebrovascular disease  **CVD risk factors:** None  **Non-cardiac disease:** None  **Medication:** None  **Clinical assessment:** symptoms, family history of CVD or cerebrovascular disease  **Blood tests:** None  **Other investigations:** None | Yes | Yes | 2 | ﻿ Inter-observer variability only | 9 |
| Li et al., 2016 | 90 | Yes | Not stated | Not stated | ﻿**Cardiac disease:** Any CVD  **CVD risk factors:** None  **Non-cardiac disease:** None  **Medication:** Any recent medications  **Clinical assessment:** Abnormal BP (90/60 mmHg–140/90  mmHg for systolic–diastolic blood pressure, respectively)  **Blood tests:** None  **Other investigations:** None | Yes | Yes | Not stated | Not stated | 8 |
| Le Ven et al., 2016 | 434 | Yes | Not stated | Phone, email, word-of-mouth invitation | ﻿**Cardiac disease:** Any CVD  **CVD risk factors:** Obesity, smoking, hyperlipidaemia, diabetes  **Non-cardiac disease:** None  **Medication:** None  **Clinical assessment:** None  **Blood tests:** Abnormal lipid profile, fasting glucose, troponin, Nt-pro-BNP  **Other investigations:** None | Yes | Yes | 4 | Yes | 9 |
| Yeon et al., 2015 | 852 | Yes | Subset of population-based study (FHS) | FHS offspring cohort who underwent CMR | ﻿**Cardiac disease:** MI, HF  **CVD risk factors:** Hypertension  **Non-cardiac disease:** None  **Medication:** Anti-hypertensives  **Clinical assessment:** BP >140/90mmHg  **Blood tests:** None  **Other investigations:** None | Yes | Yes | 1 | Not stated | 9 |
| Macedo et al., 2013 | 107 | Yes | Subset of LAC-CMR registry | Brazilian subset of LAC-CMR registry. ﻿Advertised on social networks, in participating universities and, private-owned clinics of the cities taking part in this study. | ﻿**Cardiac disease:** Any cardiomyopathy  **CVD risk factors:** Hypertension, current/ex- smoker, diabetes  **Non-cardiac disease:** None  **Medication:** Anti-hypertensives  **Clinical assessment:** BP (systolic > 120 mmHg or diastolic > 80 mmHg), symptoms, abnormal physical examination  **Blood tests:** fasting glycemia > 100 mg/dL, total cholesterol > 200 mg/dL, abnormal BNP  **Other investigations:** Abnormal ECG | Yes | Yes | 3 | Yes | 10 |
| Chang et al., 2012 | 124 | Yes | Not stated | Prospective recruitment | **Cardiac disease:** Any CVD, cerebrovascular disease  **CVD risk factors:** Hypertension, diabetes  **Non-cardiac disease:** None  **Medication:** Any regular medications  **Clinical assessment:** History of chest pain or dyspnoea  **Blood tests:** None  **Other investigations:** Abnormal echo/ECG | Yes | Yes | 1 | Yes | 8 |
| Teo et al., 2008 | 60 | Yes | Not stated | Consecutive recruitment | **Cardiac disease:** Any CVD  **CVD risk factors:** Hypertension  **Non-cardiac disease:** Respiratory disease  **Medication:** Any regular medications  **Clinical assessment:** normal BP  **Blood tests:** None  **Other investigations:** Abnormal echo/ECG﻿ | Yes | Yes | 1 | Yes | 8 |
| Maceira et al., 2006 | 120 | Yes | Not stated | Not stated | ﻿**Cardiac disease:** Any CVD  **CVD risk factors:** “Any known risk factors”  **Non-cardiac disease:** None  **Medication:** None  **Clinical assessment:** Symptoms, abnormal physical examination  **Blood tests:** Abnormal BNP  **Other investigations:** Abnormal ECG﻿ | Yes | Yes | Not stated | Not stated | 8 |
| Nikitin et al., 2006 | 95 | Yes | Primary care practice lists | All individuals without chronic illness, CVD, or regular medication were invited | **Cardiac disease:** Any CVD  **CVD risk factors:** Hypertension  **Non-cardiac disease:** Any chronic illness  **Medication:** None  **Clinical assessment:** Blood pressure >160/95, BMI >30kg/m^2^  **Blood tests:** fasting ﻿blood glucose >105 mg/dl  **Other investigations:**  Positive treadmill exercise test, Abnormal echo | Yes | Yes | Not stated | Not stated | 8 |
| Hudsmith et al., 2005 | 108 | Yes | Not stated | Not stated | **Cardiac disease:** Any CVD  **CVD risk factors:** Hypertension, “cardiac risk factors”  **Non-cardiac disease:** None  **Medication:** None  **Clinical assessment:** None  **Blood tests:** None  **Other investigations:** Abnormal ECG | Yes | Yes | 2 | Yes | 7 |
| Alfakih et al., 2003 | 60 | Yes | Not stated | Not stated | ﻿ **Cardiac disease:** Any CVD  **CVD risk factors:** diabetes, Hypertension  **Non-cardiac disease:** None  **Medication:** None  **Clinical assessment:** Abnormal cardiac examination, abnormal BP  **Blood tests:** None  **Other investigations:** Abnormal ECG | Yes | Yes | 1 | Yes | 7 |

LAC-CMR: ﻿The Latin-American, Multi-Centres, reference study of CMR (CMR-LAC Registry) ECG: electrocardiography FHS: Framingham Heart Study; SHIP: ﻿Study of Health in Pomerania; UKB: United Kingdom Biobank; CVD: cardiovascular disease; MI: myocardial infarction; ICC: intra-class correlation; COV: coefficient of variation; CMR: cardiac magnetic resonance; HF: heart failure; PVD: peripheral vascular disease; Nt-ProBNP: N terminal pro B-Type Natriuretic Peptide; *Max score =10/10
