# Supplementary material for: Variation in left ventricular cardiac magnetic resonance normal reference ranges: systematic review and meta-analysis
Source: Eur Heart J Cardiovasc Imaging. 2020 May 27;22(5):494–504. doi: 10.1093/ehjci/jeaa089 (PMC8081427; doi:10.1093/ehjci/jeaa089)
Supplement: jeaa089_Supplementary_Data [file jeaa089_supplementary_data.zip › Supp_Table3_ehj.docx]

**Supplementary Table 3. Pooled mean left ventricular parameters stratified by sex and age with expression of subgroup heterogeneity**

|  |  | Age group (years) | *n^*^* | Mean | 95% CI | subgroup heterogeneity | | | |
| --- | --- | --- | --- | --- | --- | --- | --- | --- | --- |
|  |  |  |  |  |  | Mean difference (20-40 years vs. >65 years) | Q statistic | p-value |  |
| LVEDVi (ml/m^2^) | Women | 20-40 | 270 | 79.0 | (76.8 – 81.2) | 14.0 | 18.9 | 8.02$\times$10^-5^ |  |
|  |  | 40-65 | 880 | 69.7 | (64.5 –74.9) |  |  |  |  |
|  |  | >65 | 318 | 65.0 | (56.9 – 73.2) |  |  |  |  |
|  | Men | 20-40 | 291 | 86.2 | (82.5 – 89.8) | 14.7 | 13.6 | 0.0011 |  |
|  |  | 40-65 | 727 | 78.7 | (73.4 – 83.9) |  |  |  |  |
|  |  | >65 | 247 | 71.5 | (63.8 – 79.2) |  |  |  |  |
| LVESVi (ml/m^2^) | Women | 20-40 | 257 | 28.7 | (26.3–31.1) | 6.7 | 5.0 | 0.081 |  |
|  |  | 40-65 | 863 | 25.4 | (21.3–29.5) |  |  |  |  |
|  |  | >65 | 318 | 22.0 | (16.0–28.1) |  |  |  |  |
|  | Men | 20-40 | 278 | 32.6 | (28.5–36.7) | 6.7 | 3.0 | 0.22 |  |
|  |  | 40-65 | 710 | 30.1 | (25.3–35.0) |  |  |  |  |
|  |  | >65 | 247 | 25.9 | (19.5–32.3) |  |  |  |  |
| LVMi (g/m^2^) | Women | 20-40 | 270 | 50.2 | (44.2–56.2) | 1.4 | 2.7 | 0.26 |  |
|  |  | 40-65 | 880 | 45.1 | (41.5–48.7) |  |  |  |  |
|  |  | >65 | 318 | 48.8 | (44.0–53.7) |  |  |  |  |
|  | Men | 20-40 | 291 | 64.3 | (57.8–70.8) | 4.5 | 1.0 | 0.60 |  |
|  |  | 40-65 | 727 | 60.9 | (56.5–65.3) |  |  |  |  |
|  |  | >65 | 247 | 59.8 | (53.0–66.6) |  |  |  |  |
| LVEF (%) | Women | 20-40 | 270 | 63.9 | (61.0–66.9) | -2.7 | 0.9 | 0.64 |  |
|  |  | 40-65 | 880 | 63.6 | (60.5–66.7) |  |  |  |  |
|  |  | >65 | 318 | 66.6 | (61.1–72.2) |  |  |  |  |
|  | Men | 20-40 | 291 | 62.4 | (58.8–66.0) | -2.1 | 0.5 | 0.77 |  |
|  |  | 40-65 | 727 | 62.2 | (59.0–65.5) |  |  |  |  |
|  |  | >65 | 247 | 64.5 | (59.0–70.0) |  |  |  |  |

CI: confidence interval; LVEDVi: left ventricular end diastolic volume indexed to body surface area; LVESVi: left ventricular end systolic volume indexed to body surface area; LVMi: left ventricular mass indexed to body surface area; LVEF: left ventricular ejection fraction. ^*^*n* denotes total number of participants in the pool. Results are from the random effects model.
