# Supplementary material for: Variation in left ventricular cardiac magnetic resonance normal reference ranges: systematic review and meta-analysis
Source: Eur Heart J Cardiovasc Imaging. 2020 May 27;22(5):494–504. doi: 10.1093/ehjci/jeaa089 (PMC8081427; doi:10.1093/ehjci/jeaa089)
Supplement: jeaa089_Supplementary_Data [file jeaa089_supplementary_data.zip › Supp_table5_ehj.docx]

**Supplementary Table 5. Sensitivity analysis for LV parameters according to scanner vendor, field strength, post-processing software, and papillary muscle contouring**

|  | Sensitivity analyses for between group differences^*^ | | | | | | | | | | | | | |
| --- | --- | --- | --- | --- | --- | --- | --- | --- | --- | --- | --- | --- | --- | --- |
|  |  | Scanner vendor | | Magnet strength | | Post-processing software | | Contouring methodology | | | Age | | Ethnicity | |
|  |  | Q | p-value | Q | p-value | Q | p-value | Q | p-value | Q | | p-value | Q | p-value |
| **LVEDVi (ml/m^2^)** | Men | 0.44 | 0.51 | 0.47 | 0.49 | 5.01 | 0.29 | 0.19 | 0.67 | **13.62** | | **0.0011** | 0.73 | 0.39 |
|  | Women | 0.28 | 0.60 | 0.22 | 0.64 | 1.80 | 0.77 | 0.43 | 0.51 | **18.86** | | **8.02**$\boldsymbol{\times}$**10^-5^** | 0.15 | 0.69 |
| **LVESVi (ml/m^2^)** | Men | 0.44 | 0.51 | 0.03 | 0.86 | **27.36** | **1.14**$\boldsymbol{\times}$**10^-6^** | 0.85 | 0.36 | 3.01 | | 0.22 | 0.3 | 0.58 |
|  | Women | 0.7 | 0.40 | 0.01 | 0.94 | **10.87** | **0.012** | 0.2 | 0.66 | 5.04 | | 0.081 | 0.03 | 0.86 |
| **LVMi (g/m^2^)** | Men | 0.28 | 0.60 | **26.55** | **2.6** $\boldsymbol{\times}$**10^-7^** | **183.48** | **1.6**$\boldsymbol{\times}$**10^-39^** | **5.71** | **0.017** | 1.02 | | 0.60 | **15.88** | **6.7**$\boldsymbol{\times}$**10^-5^** |
|  | Women | 1.03 | 0.31 | **4.28** | **0.039** | **8.09** | **0.044** | **4.77** | **0.029** | 2.68 | | 0.26 | **5.85** | **0.016** |
| **LVEF (%)** | Men | 0.45 | 0.50 | 0.07 | 0.80 | 6.49 | 0.17 | 1.41 | 0.23 | 0.53 | | 0.77 | 0 | 0.95 |
|  | Women | 0.71 | 0.40 | 0.02 | 0.89  ^*^scanner vendor (Siemens, Philips), field strength (1.5T, 3T), post-processing software [CMR42 (Circle Cardiovascular imaging, Qmass (Medis), MRI-mass (Medis), Argus(Siemens)], and contouring methodology (papillary muscles included vs excluded from LVM). CI: confidence interval; LVEDVi: left ventricular end diastolic volume indexed to body surface area (ml/m^2^); LVESVi: left ventricular end systolic volume indexed to body surface area (ml/m^2^); LVMi: left ventricular mass indexed to body surface area (g/m^2^); LVEF: left ventricular ejection fraction (%). Results are based on random effects estimates. significance level is set at p-value <0.05. | 6.51 | 0.16 | 0.53 | 0.47 | 0.91 | | 0.64 | 0.1 | 0.75 |
