# Supplementary material for: Variation in left ventricular cardiac magnetic resonance normal reference ranges: systematic review and meta-analysis
Source: Eur Heart J Cardiovasc Imaging. 2020 May 27;22(5):494–504. doi: 10.1093/ehjci/jeaa089 (PMC8081427; doi:10.1093/ehjci/jeaa089)
Supplement: jeaa089_Supplementary_Data [file jeaa089_supplementary_data.zip › Suppl_Table4_ehj.docx]

**Supplementary Table 4. Pooled mean left ventricular parameters with sex and ethnicity stratification and expression of subgroup heterogeneity**

|  |  | Ethnicity | *n* | Mean (95% CI)^*^ | Subgroup heterogeneity | | |
| --- | --- | --- | --- | --- | --- | --- | --- |
|  |  |  |  |  | Mean difference | Q statistic | p-value |
| LVEDVi (ml/m^2^) | Women | Caucasian | 1,825 | 71.7 (67.2–76.2) | 3.2 | 1.13 | 0.57 |
|  |  | East Asian | 254 | 70.8 (69.6–72.0) |  |  |  |
|  |  | Brazilian^*^ | 53 | 68.5 (64.3–72.2) |  |  |  |
|  | Men | Caucasian | 1,576 | 78.4 (73.5–83.2) | 4.2 | 1.84 | 0.40 |
|  |  | East Asian | 260 | 75.6 (71.5–79.7) |  |  |  |
|  |  | Brazilian | 54 | 74.2 (70.1–78.4) |  |  |  |
| LVESVi (ml/m^2^) | Women | Caucasian | 1776 | 24.5 (21.4– 27.7) | 0.5 | 0.06 | 0.97 |
|  |  | East Asian | 254 | 24.2 (22.5–26.0) |  |  |  |
|  |  | Brazilian | 53 | ﻿24.0 (21.4–27.6) |  |  |  |
|  | Men | Caucasian | 1,576 | 29.1 (25.2–33.0) | 2.8 | 1.61 | 0.45 |
|  |  | East Asian | 260 | 27.4 (23.0–31.9) |  |  |  |
|  |  | Brazilian | 54 | ﻿26.3 (24.5–28.7) |  |  |  |
| LVMi (g/m^2^) | Women | Caucasian | 1,825 | 50.6 (47.2–54.0) | 6.4 | 8.37 | 0.015 |
|  |  | East Asian | 254 | 44.2 (40.3–48.1) |  |  |  |
|  |  | Brazilian | 53 | 44.6 (40.5–48.5) |  |  |  |
|  | Men | Caucasian | 1,576 | 63.4 (59.3–67.4) | 9.8 | 18.76 | 8.44 $\times$10^-5^ |
|  |  | East Asian | 260 | 53.6 (51.0–56.2) |  |  |  |
|  |  | Brazilian | 54 | 59.8 (55.6–63.2) |  |  |  |
| LVEF (%) | Women | Caucasian | 1,825 | 65.0 (62.5–67.5) | 1.1 | 0.5 | 0.78 |
|  |  | East Asian | 254 | 65.6 (63.0–68.2) |  |  |  |
|  |  | Brazilian | 53 | 66.1 (64.9–68.6) |  |  |  |
|  | Men | Caucasian | 1,576 | 63.8 (60.9–66.7) | 1.3 | 0.62 | 0.73 |
|  |  | East Asian | 260 | 63.6 (59.3–67.9) |  |  |  |
|  |  | Brazilian | 54 | 64.9 (63.0–66.7) |  |  |  |

CI: confidence interval; LVEDVi: left ventricular end-diastolic volume indexed to body surface area (ml/m^2^); LVESVi: left ventricular end-systolic volume indexed to body surface area (ml/m^2^); LVMi: left ventricular mass indexed to body surface area (g/m^2^); LVEF: left ventricular ejection fraction (%). Random effects estimates are presented. Asian refers to Chinese, Singaporean-Chinese, and Korean ethnicity. *The Brazilian cohort is from a single study and does not represent pooled analysis, the authors do not explicitly state ethnicity- hence we have labelled results here as “Brazilian”.
